# 2-(3-Bromo­prop­yl)isoindoline-1,3-dione

**DOI:** 10.1107/S1600536809038021

**Published:** 2009-10-03

**Authors:** Peng-Fei Cheng, Chao-Jie Wang, Yu-Xia Wang

**Affiliations:** a College of Chemistry and Chemical Engineering, Henan University, Kaifeng 475004, People’s Republic of China; b Key Laboratory of Natural Medicine and Immune Engineering, Henan University, Kaifeng 475004, People’s Republic of China

## Abstract

In the title compound, C_11_H_10_BrNO_2_, the dihedral angle between the five- and six-membered rings of the phthalamide system is 1.00 (16)°. There are no significant inter­molecular inter­ations except for van der Waals contacts.

## Related literature

For pharmacological background on phthalamides, see: Brańa & Ramos (2001 ▶).
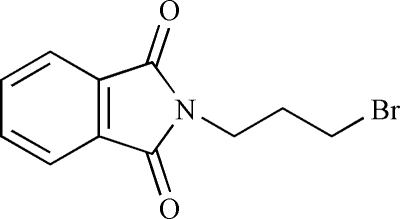


## Experimental

### 

#### Crystal data

C_11_H_10_BrNO_2_
*M* *_r_* = 268.11
Monoclinic, 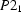

*a* = 4.8413 (7) Å
*b* = 7.3401 (11) Å
*c* = 15.095 (2) Å
β = 91.729 (3)°
*V* = 536.18 (14) Å^3^
*Z* = 2
Mo *K*α radiation
μ = 3.81 mm^−1^
*T* = 296 K
0.37 × 0.35 × 0.29 mm

#### Data collection

Bruker SMART CCD diffractometer
Absorption correction: multi-scan (*SADABS*; Bruker, 2001 ▶) *T*_min_ = 0.333, *T*_max_ = 0.405
2879 measured reflections
1888 independent reflections
1622 reflections with *I* > 2σ(*I*)
*R*_int_ = 0.018

#### Refinement

*R*[*F* ^2^ > 2σ(*F* ^2^)] = 0.026
*wR*(*F* ^2^) = 0.060
*S* = 1.00
1888 reflections
136 parameters
1 restraint
H-atom parameters constrained
Δρ_max_ = 0.41 e Å^−3^
Δρ_min_ = −0.25 e Å^−3^
Absolute structure: Flack (1983 ▶), 763 Friedel pairs
Flack parameter: 0.047 (11)

### 

Data collection: *SMART* (Bruker, 2001 ▶); cell refinement: *SAINT-Plus* (Bruker, 2001 ▶); data reduction: *SAINT-Plus*; program(s) used to solve structure: *SHELXS97* (Sheldrick, 2008 ▶); program(s) used to refine structure: *SHELXL97* (Sheldrick, 2008 ▶); molecular graphics: *PLATON* (Spek, 2009 ▶); software used to prepare material for publication: *PLATON*.

## Supplementary Material

Crystal structure: contains datablocks global, I. DOI: 10.1107/S1600536809038021/hb5110sup1.cif

Structure factors: contains datablocks I. DOI: 10.1107/S1600536809038021/hb5110Isup2.hkl

Additional supplementary materials:  crystallographic information; 3D view; checkCIF report

## Figures and Tables

**Fig. 1.**
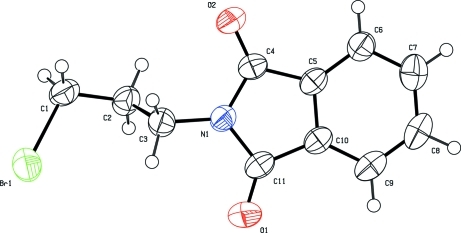
The molecular structure of (I), showing the atom-labelling scheme. Displacement ellipsoids are drawn at the 50% probability level.

## supplementary crystallographic information

### Comment

Phthalimides are well known cytotoxic DNA intercalating agents and have shown promise as potential anti-cancer agents (e.g. Brańa & Ramos, 2001). Its derivatives, such as bis-naphthalimides etc, represent a promising group of DNA-targeted anticancer agents, and the search for more potent analogues remains a priority. We now report the crystal structure of the title compound, (I).

As shown in Fig.1, the title compound consists of a phthalimide group supporting a bromopropane group. In the structure of (I), C11–O1 [1.210 (4) Å] and C4–O2 [1.208 (3) Å] are typical for a C==O double bond, the S(5) ring of N1/C4/C5/C10/C11 and the aromatic ring is approximatively coplanear, characterized by a dihedral angle of 1.00 (16)°.

### Experimental

To a mixture of 1,3-dibromopropane (46 ml, 0.45 mol) and acetone (100 ml), potassium phthalimide (22.7 g, 0.15 mol) was added in batches with refluxing. After stirring for additional 12 h, the solid was filtered off, the solvent evaporated in vacuo.  The residue was recrystallized in ethanol: evaporation gave (I) as colourless blocks (25.45 g, 63.4%).

### Refinement

H atoms were placed geometrically with C—H = 0.93–0.97Å, and refined as riding with *U*_iso_(*H*)=1.2*U*_eq_(C).

### Crystal data

**Table:**

C_11_H_10_BrNO_2_	*F*(000) = 268
*M**_r_* = 268.11	*D*_x_ = 1.661 Mg m^−^^3^
Monoclinic, *P*2_1_	Mo *K*α radiation, λ = 0.71073 Å
Hall symbol: P 2yb	Cell parameters from 1480 reflections
*a* = 4.8413 (7) Å	θ = 2.8–24.1°
*b* = 7.3401 (11) Å	µ = 3.81 mm^−^^1^
*c* = 15.095 (2) Å	*T* = 296 K
β = 91.729 (3)°	Block, colourless
*V* = 536.18 (14) Å^3^	0.37 × 0.35 × 0.29 mm
*Z* = 2	

### Data collection

**Table:**

Bruker SMART CCD diffractometer	1888 independent reflections
Radiation source: fine-focus sealed tube	1622 reflections with *I* > 2σ(*I*)
graphite	*R*_int_ = 0.018
ω scans	θ_max_ = 26.0°, θ_min_ = 2.7°
Absorption correction: multi-scan (*SADABS*; Bruker, 2001)	*h* = −5→4
*T*_min_ = 0.333, *T*_max_ = 0.405	*k* = −9→8
2879 measured reflections	*l* = −17→18

### Refinement

**Table:**

Refinement on *F*^2^	Secondary atom site location: difference Fourier map
Least-squares matrix: full	Hydrogen site location: inferred from neighbouring sites
*R*[*F*^2^ > 2σ(*F*^2^)] = 0.026	H-atom parameters constrained
*wR*(*F*^2^) = 0.060	*w* = 1/[σ^2^(*F*_o_^2^) + (0.0027*P*)^2^] where *P* = (*F*_o_^2^ + 2*F*_c_^2^)/3
*S* = 1.00	(Δ/σ)_max_ = 0.001
1888 reflections	Δρ_max_ = 0.41 e Å^−^^3^
136 parameters	Δρ_min_ = −0.25 e Å^−^^3^
1 restraint	Absolute structure: Flack (1983), 763 Friedel pairs
Primary atom site location: structure-invariant direct methods	Flack parameter: 0.047 (11)

### Special details

**Table:**

Geometry. All e.s.d.'s (except the e.s.d. in the dihedral angle between two l.s. planes) are estimated using the full covariance matrix. The cell e.s.d.'s are taken into account individually in the estimation of e.s.d.'s in distances, angles and torsion angles; correlations between e.s.d.'s in cell parameters are only used when they are defined by crystal symmetry. An approximate (isotropic) treatment of cell e.s.d.'s is used for estimating e.s.d.'s involving l.s. planes.
Refinement. Refinement of *F*^2^ against ALL reflections. The weighted *R*-factor *wR* and goodness of fit *S* are based on *F*^2^, conventional *R*-factors *R* are based on *F*, with *F* set to zero for negative *F*^2^. The threshold expression of *F*^2^ > σ(*F*^2^) is used only for calculating *R*-factors(gt) *etc*. and is not relevant to the choice of reflections for refinement. *R*-factors based on *F*^2^ are statistically about twice as large as those based on *F*, and *R*- factors based on ALL data will be even larger.

### Fractional atomic coordinates and isotropic or equivalent isotropic displacement parameters (Å^2^)

**Table:**

	*x*	*y*	*z*	*U*_iso_*/*U*_eq_	
N1	0.6038 (5)	0.5266 (4)	0.75371 (15)	0.0421 (6)	
Br1	0.85905 (7)	−0.00237 (6)	0.598616 (19)	0.06409 (14)	
O1	0.9554 (5)	0.4693 (4)	0.85776 (14)	0.0650 (7)	
O2	0.2346 (5)	0.6583 (3)	0.67773 (15)	0.0544 (6)	
C1	0.6620 (7)	0.2145 (5)	0.5540 (2)	0.0525 (8)	
H1A	0.7194	0.2405	0.4943	0.063*	
H1B	0.4651	0.1897	0.5513	0.063*	
C2	0.7160 (7)	0.3787 (4)	0.61137 (19)	0.0492 (8)	
H2A	0.6288	0.4842	0.5838	0.059*	
H2B	0.9134	0.4012	0.6154	0.059*	
C3	0.6075 (7)	0.3556 (4)	0.7045 (2)	0.0489 (8)	
H3A	0.4215	0.3064	0.7004	0.059*	
H3B	0.7228	0.2685	0.7368	0.059*	
C4	0.4095 (6)	0.6634 (4)	0.7365 (2)	0.0415 (7)	
C5	0.4650 (6)	0.8063 (4)	0.80381 (19)	0.0398 (7)	
C6	0.3321 (6)	0.9709 (5)	0.8172 (2)	0.0480 (8)	
H6A	0.1856	1.0083	0.7803	0.058*	
C7	0.4252 (8)	1.0770 (5)	0.8873 (2)	0.0567 (9)	
H7A	0.3434	1.1896	0.8971	0.068*	
C8	0.6392 (7)	1.0182 (7)	0.94342 (19)	0.0571 (9)	
H8A	0.6951	1.0907	0.9912	0.068*	
C9	0.7708 (7)	0.8541 (5)	0.9298 (2)	0.0514 (8)	
H9A	0.9156	0.8158	0.9672	0.062*	
C10	0.6813 (6)	0.7490 (4)	0.85924 (19)	0.0409 (7)	
C11	0.7744 (7)	0.5670 (4)	0.82773 (19)	0.0464 (8)	

### Atomic displacement parameters (Å^2^)

**Table:**

	*U*^11^	*U*^22^	*U*^33^	*U*^12^	*U*^13^	*U*^23^
N1	0.0494 (13)	0.0407 (19)	0.0357 (11)	0.0000 (12)	−0.0045 (10)	−0.0009 (12)
Br1	0.0801 (2)	0.0547 (2)	0.0575 (2)	0.0109 (2)	0.00154 (15)	−0.0067 (3)
O1	0.0724 (15)	0.0709 (18)	0.0506 (11)	0.0190 (15)	−0.0163 (11)	0.0038 (14)
O2	0.0581 (14)	0.0549 (14)	0.0491 (13)	0.0018 (11)	−0.0188 (11)	−0.0052 (11)
C1	0.061 (2)	0.054 (2)	0.0415 (17)	−0.0002 (15)	−0.0124 (16)	0.0022 (15)
C2	0.067 (2)	0.0421 (17)	0.0381 (16)	−0.0027 (15)	−0.0032 (15)	0.0027 (14)
C3	0.066 (2)	0.0390 (18)	0.0416 (16)	0.0025 (15)	−0.0022 (15)	0.0016 (14)
C4	0.0398 (17)	0.0461 (18)	0.0385 (16)	−0.0046 (14)	−0.0016 (14)	0.0050 (13)
C5	0.0417 (16)	0.0461 (17)	0.0316 (14)	−0.0081 (13)	0.0013 (12)	0.0027 (12)
C6	0.0529 (16)	0.048 (2)	0.0428 (14)	−0.0023 (16)	−0.0006 (12)	−0.0015 (17)
C7	0.069 (2)	0.0488 (18)	0.053 (2)	−0.0096 (16)	0.0114 (18)	−0.0059 (15)
C8	0.0614 (19)	0.069 (3)	0.0406 (15)	−0.019 (2)	0.0050 (14)	−0.015 (2)
C9	0.0483 (19)	0.069 (2)	0.0363 (16)	−0.0116 (17)	−0.0025 (15)	0.0002 (16)
C10	0.0424 (16)	0.0505 (17)	0.0300 (14)	−0.0065 (13)	0.0027 (13)	0.0042 (13)
C11	0.0519 (19)	0.0556 (19)	0.0314 (15)	−0.0024 (15)	−0.0024 (14)	0.0040 (13)

### Geometric parameters (Å, °)

**Table:**

N1—C4	1.395 (4)	C3—H3B	0.9700
N1—C11	1.401 (4)	C4—C5	1.479 (4)
N1—C3	1.459 (4)	C5—C10	1.386 (4)
Br1—C1	1.965 (3)	C5—C6	1.387 (5)
O1—C11	1.210 (4)	C6—C7	1.379 (5)
O2—C4	1.208 (3)	C6—H6A	0.9300
C1—C2	1.503 (4)	C7—C8	1.387 (5)
C1—H1A	0.9700	C7—H7A	0.9300
C1—H1B	0.9700	C8—C9	1.381 (6)
C2—C3	1.525 (4)	C8—H8A	0.9300
C2—H2A	0.9700	C9—C10	1.374 (4)
C2—H2B	0.9700	C9—H9A	0.9300
C3—H3A	0.9700	C10—C11	1.492 (4)
			
C4—N1—C11	112.0 (3)	N1—C4—C5	106.0 (2)
C4—N1—C3	122.9 (2)	C10—C5—C6	121.5 (3)
C11—N1—C3	124.9 (3)	C10—C5—C4	108.5 (3)
C2—C1—Br1	112.2 (2)	C6—C5—C4	130.0 (3)
C2—C1—H1A	109.2	C7—C6—C5	117.5 (3)
Br1—C1—H1A	109.2	C7—C6—H6A	121.3
C2—C1—H1B	109.2	C5—C6—H6A	121.3
Br1—C1—H1B	109.2	C6—C7—C8	120.9 (4)
H1A—C1—H1B	107.9	C6—C7—H7A	119.5
C1—C2—C3	112.6 (3)	C8—C7—H7A	119.5
C1—C2—H2A	109.1	C9—C8—C7	121.3 (3)
C3—C2—H2A	109.1	C9—C8—H8A	119.3
C1—C2—H2B	109.1	C7—C8—H8A	119.3
C3—C2—H2B	109.1	C10—C9—C8	117.9 (3)
H2A—C2—H2B	107.8	C10—C9—H9A	121.0
N1—C3—C2	112.5 (3)	C8—C9—H9A	121.0
N1—C3—H3A	109.1	C9—C10—C5	120.8 (3)
C2—C3—H3A	109.1	C9—C10—C11	131.2 (3)
N1—C3—H3B	109.1	C5—C10—C11	108.0 (3)
C2—C3—H3B	109.1	O1—C11—N1	125.2 (3)
H3A—C3—H3B	107.8	O1—C11—C10	129.3 (3)
O2—C4—N1	124.6 (3)	N1—C11—C10	105.5 (3)
O2—C4—C5	129.4 (3)		
			
Br1—C1—C2—C3	−64.1 (4)	C7—C8—C9—C10	−0.8 (5)
C4—N1—C3—C2	74.3 (4)	C8—C9—C10—C5	−0.2 (5)
C11—N1—C3—C2	−111.8 (3)	C8—C9—C10—C11	−178.9 (3)
C1—C2—C3—N1	−167.3 (3)	C6—C5—C10—C9	0.2 (4)
C11—N1—C4—O2	−178.0 (3)	C4—C5—C10—C9	−178.1 (3)
C3—N1—C4—O2	−3.4 (5)	C6—C5—C10—C11	179.1 (3)
C11—N1—C4—C5	1.7 (3)	C4—C5—C10—C11	0.9 (3)
C3—N1—C4—C5	176.3 (3)	C4—N1—C11—O1	178.3 (3)
O2—C4—C5—C10	178.1 (3)	C3—N1—C11—O1	3.8 (5)
N1—C4—C5—C10	−1.6 (3)	C4—N1—C11—C10	−1.1 (3)
O2—C4—C5—C6	0.0 (5)	C3—N1—C11—C10	−175.6 (3)
N1—C4—C5—C6	−179.6 (3)	C9—C10—C11—O1	−0.5 (5)
C10—C5—C6—C7	0.7 (4)	C5—C10—C11—O1	−179.3 (3)
C4—C5—C6—C7	178.6 (3)	C9—C10—C11—N1	178.9 (3)
C5—C6—C7—C8	−1.7 (5)	C5—C10—C11—N1	0.1 (3)
C6—C7—C8—C9	1.7 (5)		
